# Associations of multiple lifestyle behaviors with allergic disease symptoms and sensitization in 9–11-year-old Finnish children

**DOI:** 10.1186/s12887-024-05218-8

**Published:** 2024-11-19

**Authors:** Henna Peltonen, Anna Kaarina Kukkonen, Liisa Korkalo, Mikael Fogelholm, Mika J. Mäkelä, Maijaliisa Erkkola, Henna Vepsäläinen

**Affiliations:** 1 Department of Food and Nutrition, University of Helsinki, P.O. Box 66, Helsinki, FI-00014 Finland; 2 New Children’s Hospital, Pediatric Research Center, University of Helsinki and Helsinki University Hospital, Stenbäckinkatu 9, P.O. Box 347, Helsinki, FI-00029 Finland; 3 Department of Allergology, University of Helsinki and Helsinki University Hospital, P.O. Box 160, Helsinki, FI-00029 Finland

**Keywords:** Pediatrics, Childhood, Lifestyle behaviors, Allergic sensitization, Atopy, Wheezing, Hay fever, Dermatitis, Atopic eczema, Atopic dermatitis

## Abstract

**Background:**

The increase in allergic diseases in children has coincided with the westernization of lifestyles. Although clustering of modifiable lifestyles has been frequently reported in children, there is limited research on how lifestyle factors collectively contribute to allergic conditions. Our aim was to identify lifestyle clusters among Finnish school-aged children and explore their associations with the prevalence of allergic disease symptoms and sensitization.

**Methods:**

We used cross-sectional data from the international ISCOLE survey and its Finnish ancillary allergy study conducted in 2012–2013. We studied 148–461 children aged 9–11 years living in the metropolitan area of Finland. Parents completed a questionnaire on their child’s allergic disease symptoms, and specific IgE responses from blood samples were analyzed to determine allergic sensitization. Lifestyle factors considered in clustering were moderate-to-vigorous-physical activity (MVPA) and nighttime sleep recorded by accelerometers, screen time inquired via a questionnaire, and healthy and unhealthy dietary patterns from food frequency questionnaire data. Lifestyle clusters were identified using K-means cluster analysis, and their associations with allergic disease symptoms and sensitization were explored using logistic regression models.

**Results:**

Two distinct and stable clusters were identified: ‘healthier lifestyle & lower MVPA’ and ‘unhealthier lifestyle & higher MVPA’. After adjustments, children in the ‘unhealthier lifestyle & higher MVPA’ cluster did not show significantly different odds for symptoms of asthma (OR: 0.80, 95% CI: 0.46–1.37), allergic rhinitis (OR: 1.32, 95% CI: 0.77–2.24), or eczema (OR: 0.89, 95% CI: 0.43–1.77) as compared to those in the ‘healthier lifestyle & lower MVPA’ cluster. Similar results were observed for sensitization to ≥ 1 inhaled allergen (OR: 1.27, 95% CI: 0.53–3.10) and sensitization to ≥ 1 food allergen (OR: 0.91, 95% CI: 0.30–2.60).

**Conclusions:**

The results suggest that modifiable lifestyle factors may not play a significant role in allergic conditions within the examined age group. Lifestyle behaviors established in earlier childhood may serve as more credible predictors of allergic outcomes.

**Supplementary Information:**

The online version contains supplementary material available at 10.1186/s12887-024-05218-8.

## Introduction

Allergic diseases, including asthma, allergic rhinitis, and atopic eczema, are among the most common chronic conditions affecting children [1–3]. Over the past decades, their burden has been high, especially among children in higher income countries [4–6]. This has been speculated to be the result of the transition to a western lifestyle, which may have modified the ability to develop immune tolerance [7, 8]. Indeed, the globalization of unhealthy eating habits has been reported in children [9], and it is common for children not to meet the recommended intake levels of fruits and vegetables [10] or target amounts of physical activity, screen time, and nighttime sleep [11]. This makes it important to investigate the role of lifestyle factors in allergic diseases more closely.

In everyday settings, lifestyle behaviors tend to cluster among children [12]. Targeting multiple behaviors simultaneously may demonstrate synergistic effects on allergy outcomes and produce the most predictive results. To the best of our knowledge, this approach has not received much attention in previous allergy research. Two earlier studies comprising 5- to 11-year-old children applied theory-based indices of healthy lifestyle adoption, showing either a protective [13] or null association [14] with allergic disease prevalence. However, data-driven approaches, such as cluster analysis, allow both healthy and unhealthy behaviors to appear in the data and can hence provide a more objective assessment of their joint associations with allergy outcomes. Cluster analysis, in particular, is a common data-driven statistical technique used for recognizing groups of children who share similar lifestyles [12].

In the current study, our first aim was to identify distinct lifestyle clusters among 9–11-year-old Finnish children based on dietary patterns, physical activity, screen time, and nightly sleep duration. Dietary patterns represent a whole-diet approach, which enabled us to capture a broad picture of the variation in children’s dietary habits [15]. Sleep, on the other hand, has not been considered very often in earlier assessments of children’s lifestyle patterning [12]. We anticipated that the lifestyle factors discriminate children in a healthy or unhealthy direction. Secondly, we aimed to explore the associations between the identified lifestyle clusters and the prevalence of allergic disease symptoms and sensitization among the children. Allergic sensitization refers to excessive production of immunoglobulin E (IgE) upon common allergen exposure [16, 17] and is a precondition for the development of allergic diseases [18, 19]. We hypothesized that the prevalence of allergic conditions is higher in the unhealthier cluster(s) than in the healthier one(s).

## Methods

### Study design and participants

**Fig. 1:**
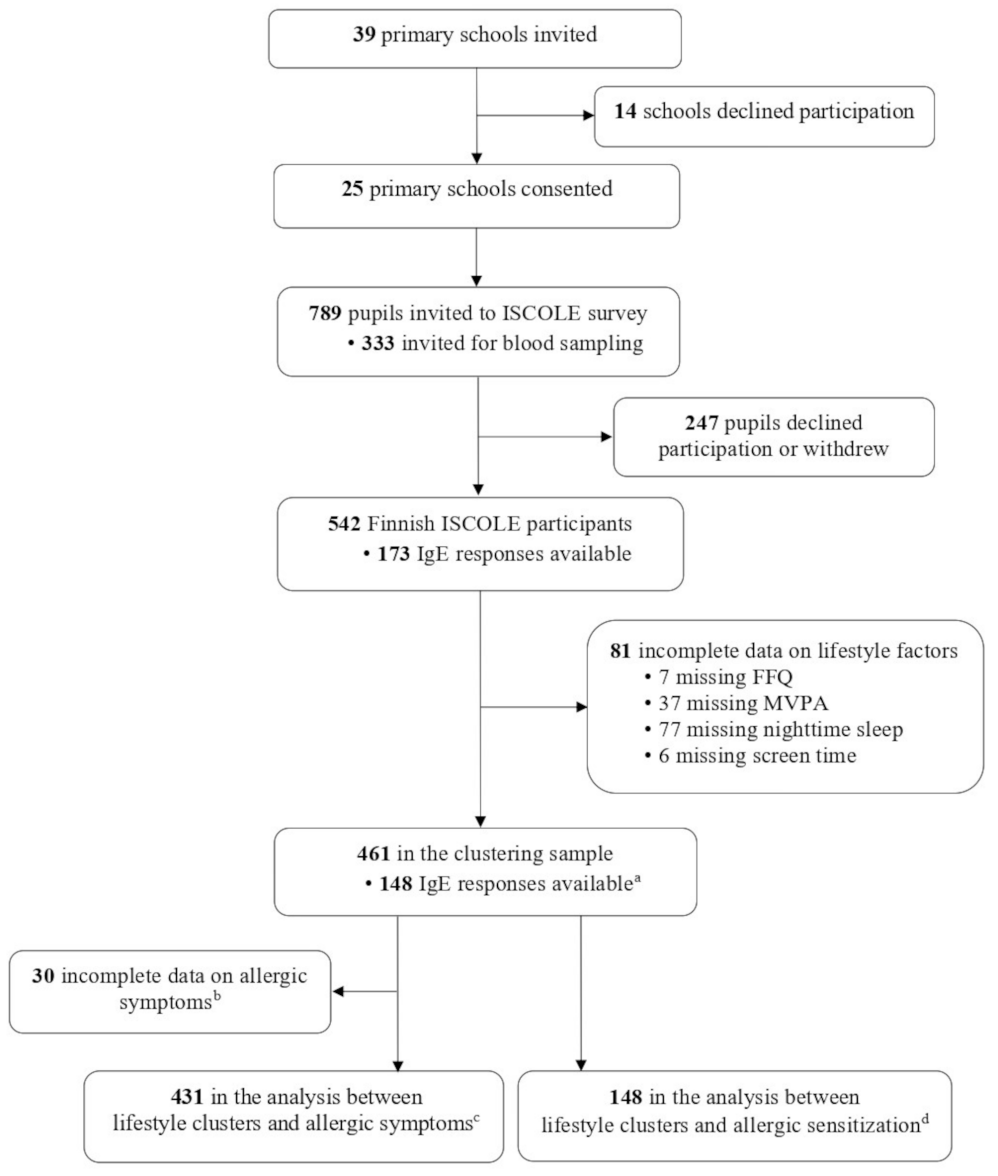
Flow chart presenting the sample sizes in the ISCOLE sub-study on lifestyles and allergic diseases ^a^ had complete data on lifestyle factors ^b^ 30 children missing asthma and eczema symptoms, 31 children missing symptoms of allergic rhinitis ^c^ primary analytic sample ^d^ secondary analytic sample Abbreviations: FFQ, food frequency questionnaire; IgE; immunoglobulin E; MVPA, moderate-to-vigorous physical activity

The current work was part of the International Study of Childhood Obesity, Lifestyle and the Environment (ISCOLE) survey, designed to examine the associations between lifestyle behaviors and obesity in children [20]. The participants were 9–11-year-old children who were recruited from schools located in urban and semi-urban areas of 12 countries, including Finland. The detailed protocol of recruitment and data collection has been published elsewhere [20]. All ISCOLE participants in Finland were also invited to the national ancillary study on allergic diseases. The current work used the data collected from Finnish ISCOLE participants alone. In brief, a cross-sectional survey was conducted between September 2012 and April 2013 in primary schools in the metropolitan area of Finland. The sampling frame comprised a complete list of primary schools of the cities of Helsinki, Espoo, and Vantaa, stratified by indicators of socio-economic status (primarily parental educational level, if available, otherwise income level). Children aged 9 to 11 years in the 4th grade were selected from each stratum of schools. One class in a school was hence the smallest unit of recruitment, and all pupils from the class were invited. Consequently, a total of 39 schools were approached, and 25 (64%) consented to participate. From these consenting schools, 789 children were invited, of whom 542 (69%) from 25 primary schools participated in the ISCOLE study in Finland (Fig. 1).

The study was conducted according to the Declaration of Helsinki and approved by the Helsinki and Uusimaa Hospital District Ethics Committees. Informed parental consent as well as child assent were obtained for each participant.

### Lifestyle factors

In the current work, we focused on the following four dimensions of lifestyle: dietary patterns, physical activity, nightly sleep duration, and screen time.

#### Dietary assessment

The children’s usual food consumption frequency was recorded using a 23-item non-quantitative food frequency questionnaire (FFQ; i.e., portion sizes were not asked), which showed acceptable validity against a pre-coded food diary [21] and reliability in assessing dietary patterns [9]. The FFQ inquired how many times children usually ate foods and beverages using the following seven frequency alternatives: never; less than once a week; once a week; 2–4 days a week; 5–6 days a week; once a day; or more than once a day.

For further analysis, consumption frequencies were recoded into numeric weekly consumption frequencies as follows: ‘never’ into 0, ‘less than once a week’ into 0.5, ‘once a week’ into 1, ‘on 2–4 days a week’ into 3, ‘on 5–6 days a week’ into 5.5, ‘once a day’ into 7, and ‘more than once a day’ into 10. Based on these numerical weekly consumption frequencies, principal component analysis (PCA) was applied to identify dietary patterns among the children. The PCA combined the consumption frequencies of foods and beverages that correlated with each other, and had been previously performed for the total international ISCOLE dataset and for the data of each country separately. In the current work, we used the country-specific dietary patterns of Finnish ISCOLE participants. Detailed description of the PCA procedures can be found elsewhere [9]. In brief, after the first run of PCA, two components were selected based on eigenvalues and scree plot inspection. Fruit juice consumption was excluded from the PCA due to its low validity [21]. A rerun with the two components was performed with an orthogonal Varimax rotation, and standardized principal component scores were computed for each child. The two components together explained 33% of the total variance in FFQ data and were labelled ‘unhealthy dietary pattern’ and ‘healthy dietary pattern’ based on the food loadings with an absolute value of > 0.30 (Supplementary Table 1, Additional File 1). The unhealthy dietary pattern was characterized by high loadings of fast foods (e.g., hamburgers, pizza), fried food (nuggets, fish sticks), French fries, potato chips, ice-cream, and sugar-sweetened sodas, whereas the healthy dietary pattern comprised high loadings for dark-green vegetables, orange vegetables, vegetables in general, fruits and berries, wholegrains, and fish.

#### Sleep duration and physical activity

Objective measurements of children’s nightly sleep duration and time spent in moderate-to-vigorous physical activity (MVPA) were obtained by 24-hour accelerometry. The children wore an Acti-Graph GT3 × 1 accelerometer (Pensacola, FL, USA) on a waistband and were encouraged to wear it 24 h per day for at least seven days, including two weekend days, while maintaining their normal daily routines.

Nightly sleep duration was estimated using a refined, validated algorithm that excluded extended episodes of nocturnal non-wear and wakefulness and avoided misclassified daytime sleep episodes [22]. Briefly, sleep time was defined as time between algorithm-determined sleep onset and sleep offset. A night was considered valid if overnight sleep time was ≥ 160 min. At least three valid nights of sleep, including at least one weekend night (Friday or Saturday), was a minimum criterion for including sleep data in the analysis. Each child’s mean nightly duration of sleep from all valid sleep nights was considered in the current analyses.

For recording physical activity, a valid day was defined as ≥ 10 h of wear time excluding sleep time and awake non-wear time (any sequence of ≥ 20 consecutive minutes of zero activity counts). At least four valid days, including at least one weekend day, were required to include physical activity data in the analysis. In the current work, we focused on MVPA since recommendations for school-aged children are based on this level of intensity [23]. Using commonly employed cut-points by Evenson et al. [24], MVPA was defined as ≥ 574 counts per 15-second period. Each child’s mean daily duration of MVPA from all valid days was considered in the current analyses.

#### Screen time

As objective measures cannot distinguish the type of sedentary behavior, we relied on the children’s own reports on their screen time. They were asked to fill in a questionnaire inquiring (1) how many hours they watched TV; and (2) how many hours they played video or computer games or used a computer for something that was not schoolwork in the last week. Both questions were asked for a typical school day and weekend day separately. Answer options were 0, < 1, 1, 2, 3, 4, and ≥ 5 h per day. To obtain mean daily screen time scores, the answer options ‘<1’ and ‘≥5’ were recoded to ‘0.5’ and ‘5’, respectively. Then, the answers on time spent with the screen devices were weighted and added up as follows: hours of TV on school days x 5/7 + hours of TV on weekend days x 2/7 + hours of video games or computer on school days x 5/7 + hours of video games or computer on weekend days x 2/7. This produced a possible range of from 0 to 10 h per day.

### Outcomes

#### Allergic disease symptoms

The prevalence of allergic symptoms, i.e., those of asthma, allergic rhinitis, and eczema, was recorded using a modified International Study of Asthma and Allergies in Childhood (ISAAC) allergy questionnaire [25] completed by the parents. The prevalence of asthma symptoms was defined as a positive answer to one or both of the following questions: ‘*Has the child had prolonged cough (for more than 6 weeks)?*’ and ‘*Has the child ever had wheezing sounds while breathing or appeared to have difficulty in breathing?*’. The prevalence of rhinitis symptoms was defined as a positive answer to one or both of the following questions: ‘*Do pollen or animals cause sneezing or runny or blocked nose?*’ and ‘*Do pollen or animals cause red*, *itchy*, *or swollen eyes?*”. The prevalence of eczema symptoms was defined as a positive answer to the question *’Does the child have dry*, *red*, *and itchy skin that requires regular care?*’.

#### IgE measurements

In order to assess allergen-specific IgE responses, blood samples were collected between January 29th and June 3rd 2013 from 173 children (52% of the invited; 32% of the Finnish survey sample) (Fig. 1). A blood specimen of 9 ml was taken from the antecubital vein. Specific IgE responses to common inhaled and food allergens were analyzed using the Pharmacia CAP-fluoroenzyme immunoassay. Measurements were performed in the laboratory of Helsinki University Hospital. Allergic sensitization was defined as an allergen-specific IgE concentration of ≥ 0.35 kU/L, based on which the following two outcomes were derived: sensitization to ≥ 1 inhaled allergen (birch, timothy grass, mugwort, cat, dog, horse, house dust mite [*Dermatophagoides Pteronyssinus*], mold [*Cladosporium Herbarum*]); and sensitization to ≥ 1 food allergen (cow’s milk, egg white, codfish, wheat, soy, peanut).

### Confounding variables

In the current work, we considered ten potential confounding variables based on theory or earlier findings. For body mass index (BMI), trained research staff measured the children’s height and weight in the schools such that the children were without shoes and heavy clothing. Height was measured using a Seca 213 portable stadiometer (Hamburg, Germany). Weight was measured using a Tanita Body Composition Analyzer SC-240 scale (Arlington Heights, Illinois). BMI was computed as kg/m^2^. Using age- and sex-specific reference data from the World Health Organization [26], BMI Z-scores were then calculated and used as a continuous variable in the current analyses. Parents reported the child’s sex and eight other background variables through questionnaires. We treated the age when solid foods were introduced and the age when completely stopped being breastfed as continuous variables. As a surrogate for the child’s birth order, we considered the number of older biological siblings the child had. This was categorized in the descriptive analyses (0, 1, ≥ 2 older siblings) and used as a count variable in the multivariable modelling. As dichotomous data, we used the information on furry pets at home or daycare facility (yes, no), maternal smoking during pregnancy (yes, no), and current parental smoking (one or both of the parents, none). Parental allergy history was defined as having a history of at least one of the following: asthma, pollen or animal allergy, food allergy, or atopic eczema. Then, parental allergy history was divided into four categories as follows: both parents, mother alone, father alone, or neither of the parents had a history of allergic disease. We divided parents’ educational level into three categories (high school or less, college, bachelor’s degree or postgraduate degree), of which the highest achieved by either parent was considered in the current work.

### Statistical methods

All analyses were carried out with two-tailed tests using the R statistical programming language version 4.3.2 [27]. We considered *P*-values below 0.05 to be statistically significant.

#### Cluster analysis

To identify groups of children with similar lifestyle behaviors in the data, a cluster analysis was employed. As input variables, we used unhealthy and healthy dietary pattern scores, MVPA, nightly sleep duration, and screen time, which were standardized into Z-scores due to different measurement units. Children who provided complete data on all five lifestyle variables were included in the analysis. Since all lifestyle variables were continuous, we considered a widely used K-means algorithm [28] to be suitable for the current work. It performs an iterative process of assigning observations to groups based on their distances from the pre-selected number of cluster centers [28]. We used the function *kmeans*, available in the R package *stats*. We set a random initial seed to initialize cluster centers and used 25 different random starting assignments to optimize the allocation to clusters of similar features. To determine the number of clusters identified, a four-cluster solution was used as a starting point, as suggested by the Elbow method, and clustering was then repeated with three- and two-cluster solutions. Distinguishing features of the cluster solutions were identified by comparing the values of the resulting cluster centers, i.e., average Z-scores. The final cluster solutions were based on interpretability and degree of distinction, inspected visually, and a cluster membership was then recorded for each child. To examine the robustness of the final cluster features, we omitted univariate outliers, i.e., those with a Z-score of > 3 or < − 3 in any of the standardized lifestyle variables, and repeated clustering. We also randomly shuffled the observation rows and repeated clustering several times to confirm whether the cluster features remained the same. Lastly, we verified the stability of the cluster solutions by randomly dividing the data into halves, i.e., two equal subsamples on which clustering was repeated. Then, Kappa degrees of agreement (Ƙ) were computed between the cluster memberships of the subsamples and those of the total sample.

#### Descriptive analyses and modelling

To compare background characteristics between the included and excluded children and across binary allergy outcomes, we used the independent samples t-test for continuous variables, Mann-Whitney U-test for skewed continuous variables, and chi-squared or Fisher’s exact test for categorical variables. A Venn diagram was drawn to illustrate the co-occurrence of allergic symptoms. Logistic regression models were used to explore the associations of lifestyle cluster memberships with the prevalence of allergic symptoms and sensitization, with results presented as odds ratios (OR) and their 95% confidence intervals (CIs). The models were fit separately for each allergy outcome. The unadjusted models included cluster membership as a predictor variable. Variables included in the adjusted models were sex, number of older siblings, parental history of allergic disease, exposure to furry pets, age when solid foods were introduced, age when breastfeeding was stopped, current parental smoking, maternal smoking during pregnancy, BMI Z-scores, and the family’s highest educational level, based on their theoretical importance. We also conducted data-driven analyses using the following exploratory outcomes in modelling: manifestation combinations of allergic symptoms (≥ 2 symptoms, all three symptoms), higher-threshold sensitization (IgE ≥ 0.70 kU/L), polysensitization (specific IgE ≥ 0.35 kU/L to two or more allergens), and symptomatic sensitization (specific IgE ≥ 0.35 kU/L to any allergen, accompanied by symptoms of any allergic disease).

## Results

Of the 542 Finnish children participating in the ISCOLE survey, 461 (85%) had complete data on lifestyle factors and were included in the cluster analysis (Fig. 1). A higher proportion of boys than girls were excluded from clustering (17 vs. 11%, *P* = 0.032), but no other differences were observed in children’s characteristics (data not shown). Among children with cluster membership, 431 (80% of all participants) had data on allergic symptoms and were included in the primary analytic sample. Additionally, among children with cluster membership, 148 children (27% of all participants; 86% of those who provided blood samples) had data on IgE responses and were included in the secondary analytic sample. Below, we describe the identified lifestyle clusters, participants of analytic samples, and the associations between lifestyle clusters and allergic outcomes.

### Description of cluster solutions

**Fig. 2:**
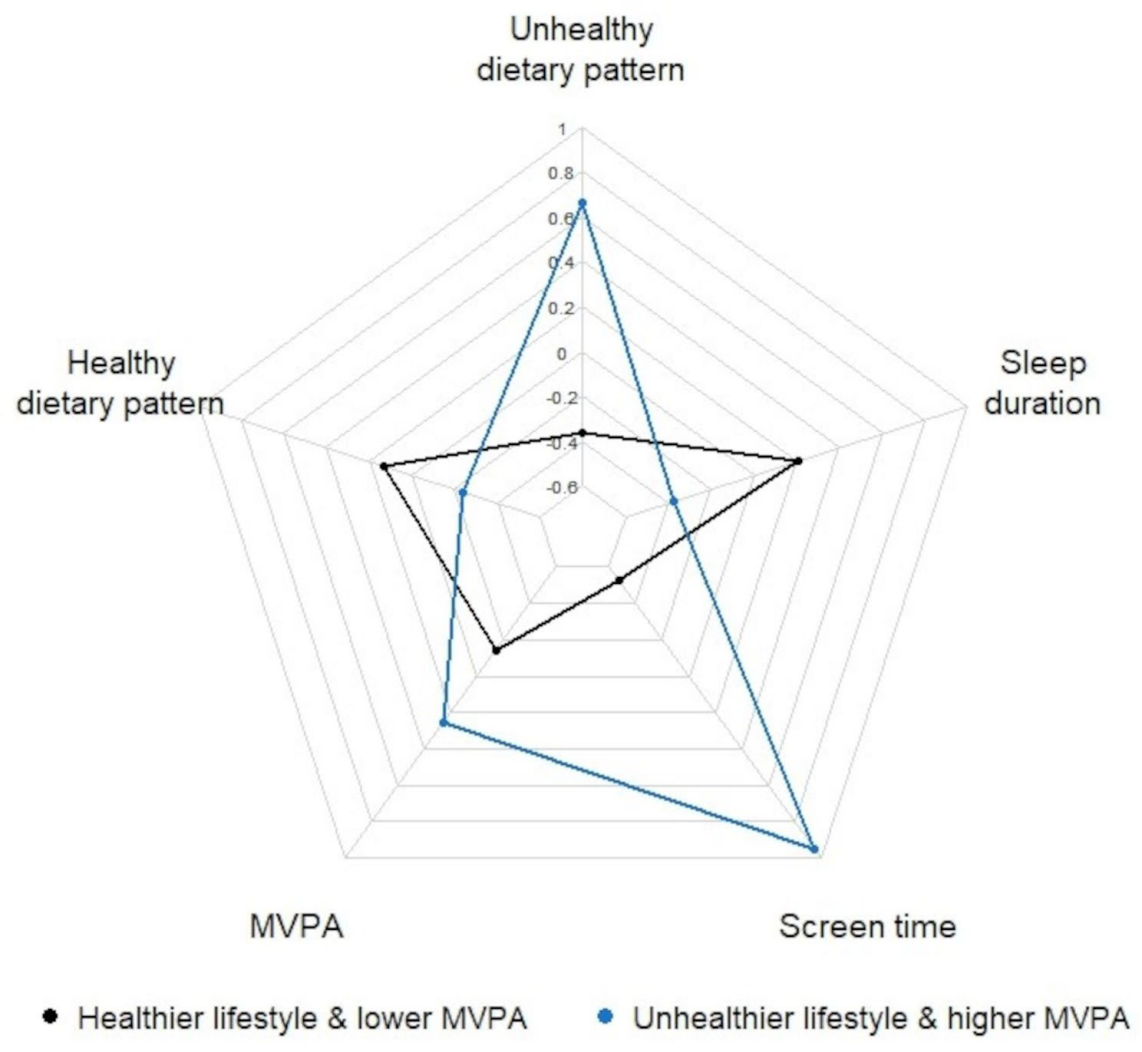
Final cluster centers (average Z-scores) of the lifestyle factors based on the K-means cluster analysis. Dietary patterns and screen time were based on self-reported data. MVPA and nightly sleep duration were based on accelerometer data. A total of 461 children were included in the analysis Abbreviations: MVPA, moderate-to-vigorous physical activity

Based on the K-means clustering, a two-cluster solution was found to be meaningful in discriminating children with complete data on the five lifestyle factors (*n* = 461). Of the children, 296 (64%) were assigned into cluster 1, scoring negatively on screen time (Z-score − 0.53), unhealthy dietary pattern (Z-score − 0.37), and MVPA (Z-score − 0.14) but positively on sleep duration (Z-score 0.21) and healthy dietary pattern (Z-score 0.13) (Fig. 2). Cluster 1 was hence labelled ‘healthier lifestyle & lower MVPA’. In turn, 165 (36%) of the children were assigned into cluster 2, scoring positively on screen time (Z-score 0.95), unhealthy dietary pattern (Z-score 0.66), and MVPA (Z-score 0.26) but negatively on sleep duration (Z-score − 0.38) and healthy dietary pattern (Z-score − 0.24). Cluster 2 was hence labelled ‘unhealthier lifestyle & higher MVPA’. The cluster features remained similar after omitting univariate outliers from the analysis (*n* = 24) or shuffling the observations rows. Stability of the cluster solutions was supported by their good agreement with the cluster solutions of random subsamples (Ƙ=0.74 and 0.88 for the two subsamples).

### Description of analytic samples

#### Primary analytic sample

**Fig. 3:**
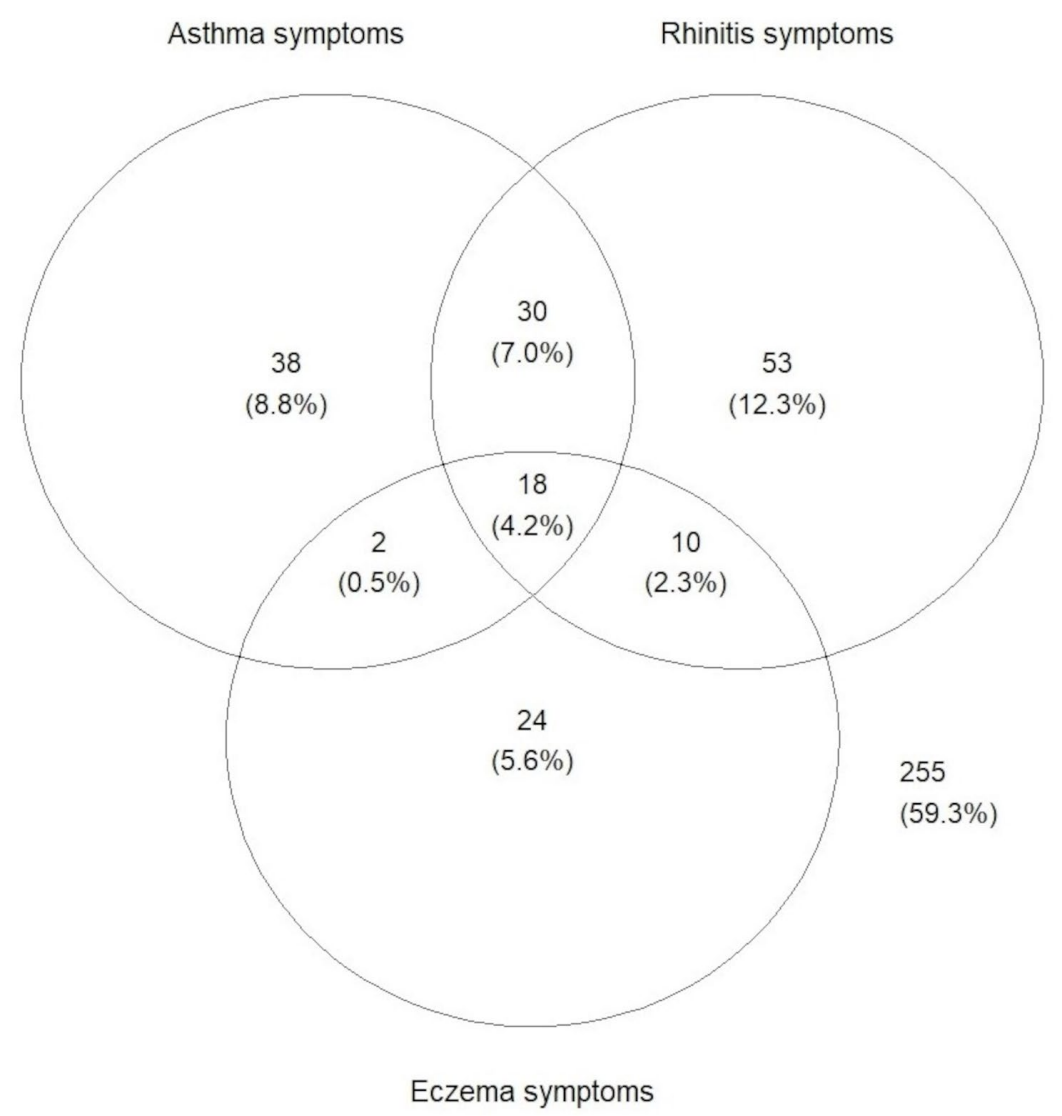
Venn diagram showing the overlap between the prevalence of allergic disease symptoms [*n*(%)]. The 255 children outside the Venn diagram represent asymptomatic children. One child had additional missing information about allergic rhinitis symptoms and could not be included in the graph, resulting in the sample size of 430 children for the analysis of co-occurring allergic disease symptoms

The prevalence of asthma, rhinitis, and eczema symptoms in the primary analytic sample (*n* = 431) was 20%, 26% and 13%, respectively. The most common manifestation combination was the co-occurrence of asthma and rhinitis symptoms (7.0%; Fig. 3).

Children with asthma symptoms had higher BMI Z-scores on average than their asymptomatic peers (0.54 ± 1.0 vs. 0.19 ± 1.0, *P* = 0.005) (Table 1). Children with rhinitis symptoms, as compared to their asymptomatic peers, had less frequent exposure to furry pets (32% vs. 49%, *P* = 0.003), a longer duration of breastfeeding (median 8 months [interquartile range (IQR) 6–12] vs. 7 [3–11], *P* = 0.021), and more often both parents with a history of allergic disease (35% vs. 9.1%, *P* < 0.001). Likewise, children with eczema symptoms had less frequent exposure to furry pets (28% vs. 47%, *P* = 0.007), a longer duration of breastfeeding (median 8 months [IQR 6–12] vs. 7 [3.9–11], *P* = 0.017), and more often both parents with a history of allergic disease (31% vs. 14%, *P* < 0.001) than asymptomatic children.

**Table 1:** Background characteristics according to the prevalence of allergic disease symptoms in the participating children

Characteristics	All	Asthma symptoms		*P*	Allergic rhinitis symptoms^a^		*P*	Eczema symptoms		*P*
		yes	no		yes	no		yes	no	
***n***	431	88	343		111	319		54	377	
**Sex**, ***n*(%)**										
Boys	196 (45)	48 (55)	148 (43)	0.055	54 (49)	141 (44)	0.418	23 (43)	173 (46)	0.649
Girls	235 (55)	40 (45)	195 (57)		57 (51)	178 (56)		31 (57)	204 (54)	
**Number of older biological siblings**, ***n*(%)**										
0	213 (49)	40 (45)	173 (50)	0.588	57 (51)	156 (49)	0.853	28 (52)	185 (49)	0.848
1	132 (31)	29 (33)	103 (30)		32 (29)	99 (31)		15 (28)	117 (31)	
≥ 2	80 (19)	19 (22)	61 (18)		22 (20)	58 (18)		11 (20)	69 (18)	
Missing	6 (1.4)	0 (0.0)	6 (1.7)		0 (0.0)	6 (1.9)		0 (0.0)	6 (1.6)	
**BMI Z-score**										
Mean ± SD	0.26 ± 1.0	**0.54 ± 1.0**	**0.19 ± 1.0**	**0.005**	0.23 ± 0.91	0.28 ± 1.1	0.660	0.21 ± 0.94	0.27 ± 1.1	0.698
Missing, *n* (%)	1 (0.23)	0 (0.0)	1 (0.29)		1 (0.90)	0 (0.0)		1 (1.9)	0 (0.0)	
**Highest educational level in the family**, ***n*(%)**										
High school or less	117 (27)	24 (27)	93 (27)	0.815	27 (24)	90 (28)	0.535	11 (20)	106 (28)	0.157
College degree	126 (29)	28 (32)	98 (29)		31 (28)	95 (30)		13 (24)	113 (30)	
Bachelor’s or postgraduate degree	187 (43)	36 (41)	151 (44)		53 (48)	133 (42)		30 (56)	157 (42)	
Missing	1 (0.23)	0 (0.0)	1 (0.29)		0 (0.0)	1 (0.31)		0 (0.0)	1 (0.27)	
**Furry pets at home or daycare facility**, ***n*(%)**										
Yes	192 (45)	34 (39)	158 (46)	0.180	**36 (32)**	**156 (49)**	**0.003**	**15 (28)**	**177 (47)**	**0.007**
Missing	4 (0.93)	0 (0.0)	4 (1.2)		1 (0.90)	3 (0.94)		0 (0.0)	4 (1.1)	
**Age at introducing solids**, **months**										
Median (IQR)	4 (3–4.5)	4 (3.5–4)	4 (3–4.5)	0.435^b^	4 (3.25–4.25)	4 (3–4.75)	0.438^b^	4 (3–4)	4 (3–4.5)	0.785^b^
Missing, *n* (%)	17 (3.9)	3 (3.4)	14 (4.1)		4 (3.6)	12 (3.8)		2 (3.7)	15 (4.0)	
**Age completely stopped being breastfed**, **months**										
Median (IQR)	7 (4–11)	8 (4–12)	7 (4–11)	0.244^b^	**8 (6–12)**	**7 (3–11)**	**0.021** ^b^	**8 (6–12)**	**7 (3.9–11)**	**0.017** ^b^
Missing, *n* (%)	9 (2.1)	1 (1.1)	8 (2.3)		1 (0.90)	7 (2.2)		0 (0.0)	9 (2.4)	
**Parental history of allergic disease**^**c**^, ***n*(%)**										
Neither parent	180 (42)	28 (32)	152 (44)	0.146	**23 (21)**	**156 (49)**	**< 0.001**	**13 (24)**	**167 (44)**	**< 0.001**
Father alone	77 (18)	21 (24)	56 (16)		**18 (16)**	**59 (19)**		**7 (13)**	**70 (19)**	
Mother alone	105 (24)	23 (26)	82 (24)		**31 (28)**	**74 (23)**		**17 (31)**	**88 (23)**	
Both parents	68 (16)	16 (18)	52 (15)		**39 (35)**	**29 (9.1)**		**17 (31)**	**51 (14)**	
Missing	1 (0.23)	0 (0.0)	1 (0.29)		0 (0.0)	1 (0.31)		0 (0.0)	1 (0.27)	
**Current parental smoking**^**d**^, ***n*(%)**										
Yes	86 (20)	22 (25)	64 (19)	0.189	25 (23)	61 (19)	0.449	7 (13)	79 (21)	0.167
Missing	1 (0.23)	0 (0.0)	1 (0.29)		0 (0.0)	1 (0.31)		0 (0.0)	1 (0.27)	
**Maternal smoking during pregnancy**, ***n*(%)**										
Yes	22 (5.1)	4 (4.5)	18 (5.2)	1.00^e^	5 (4.5)	17 (5.3)	0.808^e^	3 (5.6)	19 (5.0)	0.751^e^
Missing	6 (1.4)	0 (0.0)	6 (1.7)		0 (0.0)	6 (1.9)		0 (0.0)	6 (1.6)	

*P*-values are based on independent samples t-tests for continuous variables and chi-squared independence tests for categorical variables, unless otherwise indicated. Significant differences across outcome categories (*P* < 0.05) are indicated in boldface. Missing observations are shown if they were present

^a^ one missing value

^b^ based on Mann-Whitney U-test

^c^ at least one of the following: asthma, pollen or animal allergy, food allergy, or atopic eczema

^d^ one or both of the parents

^e^ based on Fisher’s exact test

Abbreviations: IQR, inter-quartile range (25th to 75th percentile); SD, standard deviation

Compared to the ‘healthier lifestyle & lower MVPA’ cluster, the ‘unhealthier lifestyle & higher MVPA’ cluster included more boys (35% vs. 64%, *P* < 0.001) but had fewer children from families with the highest educational level (47% vs. 37%, *P* = 0.022) (Supplementary Table 2, Additional File 1).

#### Secondary analytic sample

Among children included in the secondary analytic sample (*n* = 148), 39% were sensitized to ≥ 1 inhaled allergen and 19% were sensitized to ≥ 1 food allergen. Prevalence of allergen-specific sensitization is shown in Supplementary Table 3, Additional File 1.

Majority of the sensitized children had at least one allergic disease symptom, with rhinitis symptoms being the most common (Table 2). Children sensitized to ≥ 1 inhaled allergen more often had both parents with a history of allergic disease as compared to their non-sensitized peers (33% vs. 7.8%, *P* < 0.001) (Table 3). Sensitization to ≥ 1 food allergen did not show significant associations with background characteristics.

**Table 2:** Prevalence of allergic disease symptoms among sensitized children

Condition	Sensitized to ≥ 1 inhaled allergen ^a^ (*n* = 58)	Sensitized to ≥ 1 food allergen ^b^ (*n* = 28)
Any allergic disease symptom, *n* (%)	44 (76)	20 (71)
Only asthma symptoms, *n* (%)	18 (31)	10 (36)
Only allergic rhinitis symptoms, *n* (%)	39 (67)	17 (61)
Only eczema symptoms, *n* (%)	13 (22)	8 (29)
No allergic disease symptoms, *n* (%)	12 (21)	6 (21)
Missing, *n* (%) ^c^	2 (3.4)	2 (7.1)

^a^ IgE response ≥ 0.35 kU/L to at least one of the following: birch, timothy grass, mugwort, cat, dog, horse, house dust mite (*Dermatophagoides Pteronyssinus*), mold (*Cladosporium Herbarum*)

^b^ IgE response ≥ 0.35 kU/L to at least one of the following: cow’s milk, egg, codfish, wheat, soy, peanut

^c^ sensitized children missing data on allergic disease symptoms

Abbreviations: IgE, Immunoglobulin E; ISCOLE, the International Study of Childhood Obesity, Lifestyle and the Environment

**Table 3:** Background characteristics according to the prevalence of allergic sensitization in the participating children

Characteristics	All	Sensitized to ≥ 1 inhaled allergen		*P*	Sensitized to ≥ 1 food allergen		*P*
		yes	no		yes	no	
***n***	148	58	90		28	120	
**Sex**, ***n*(%)**							
Boys	70 (47)	29 (50)	41 (46)	0.597	11 (39)	59 (49)	0.346
Girls	78 (53)	29 (50)	49 (54)		17 (61)	61 (51)	
**Number of older biological siblings**, ***n*(%)**							
0	65 (44)	24 (41)	41 (46)	0.680	13 (46)	52 (43)	0.921^a^
1	47 (32)	20 (34)	27 (30)		10 (36)	37 (31)	
≥ 2	26 (18)	12 (21)	14 (16)		4 (14)	22 (18)	
Missing	10 (6.8)	2 (3.4)	8 (8.9)		1 (3.6)	9 (7.5)	
**BMI Z-score**							
Mean ± SD	0.33 ± 1.1	0.23 ± 1.1	0.39 ± 1.1	0.379	0.36 ± 1.1	0.32 ± 1.1	0.863
**Highest educational level in the family**, ***n*(%)**							
High school or less	35 (24)	11 (19)	24 (27)	0.240	5 (18)	30 (25)	0.637^a^
College degree	33 (22)	17 (29)	16 (18)		6 (21)	27 (23)	
Bachelor’s or postgraduate degree	72 (49)	29 (50)	43 (48)		16 (57)	56 (47)	
Missing	8 (5.4)	1 (1.7)	7 (7.8)		1 (3.6)	7 (5.8)	
**Furry pets at home or daycare facility**, ***n*(%)**							
Yes	53 (36)	17 (29)	36 (40)	0.121	7 (25)	46 (38)	0.192
Missing	9 (6.1)	2 (3.4)	7 (7.8)		2 (7.1)	7 (5.8)	
**Age at introducing solids**, **months**							
Median (IQR)	4 (3–4.5)	4 (3–4)	4 (3.25–4.5)	0.609^b^	4 (4–4)	4 (3–4.5)	0.892^b^
Missing, *n* (%)	16 (11)	4 (6.9)	12 (13)		3 (11)	13 (11)	
**Age completely stopped being breastfed**, **months**							
Median (IQR)	8 (5–12)	8 (4.5–12)	8 (5.5–12)	0.869^b^	6.75 (4.25–11)	8 (5–12)	0.161^b^
Missing, *n* (%)	12 (8.1)	3 (5.2)	9 (10)		2 (7.1)	10 (8.3)	
**Parental history of allergic disease**^**c**^, ***n*(%)**							
Neither parent	53 (36)	**14 (24)**	**39 (43)**	**< 0.001**	10 (36)	43 (36)	0.231^a^
Father alone	28 (19)	**9 (16)**	**19 (21)**		5 (18)	23 (19)	
Mother alone	32 (22)	**14 (24)**	**18 (20)**		3 (11)	29 (24)	
Both parents	26 (18)	**19 (33)**	**7 (7.8)**		8 (29)	18 (15)	
Missing	9 (6.1)	2 (3.4)	7 (7.8)		2 (7.1)	7 (5.8)	
**Current parental smoking**^**d**^, ***n*(%)**							
Yes	26 (18)	10 (17)	16 (18)	0.833	5 (18)	21 (18)	1.00^a^
Missing	9 (6.1)	2 (3.4)	7 (7.8)		2 (7.1)	7 (5.8)	
**Maternal smoking during pregnancy**, ***n*(%)**							
Yes	5 (3.4)	2 (3.4)	3 (3.3)	1.00^a^	1 (3.6)	4 (3.3)	1.00^a^
Missing	12 (8.1)	3 (5.2)	9 (10)		2 (7.1)	10 (8.3)	

*P*-values are based on independent samples t-tests for continuous variables and chi-squared independence tests for categorical variables unless otherwise indicated. Significant differences across outcome categories (*P* < 0.05) are indicated in boldface. Missing observations are shown if they were present

^a^ based on Fisher’s exact test

^b^ based on Mann-Whitney U-test

^c^ at least one of the following: asthma, pollen or animal allergy, food allergy, or atopic eczema

^d^ one or both of the parents

Abbreviations: IQR, inter-quartile range (25th to 75th percentile); SD, standard deviation

The significant sex difference between the lifestyle clusters persisted in the secondary analytic sample (61% in the ‘unhealthier lifestyle & higher MVPA’ cluster were boys vs. 40% in the ‘healthier lifestyle & lower MVPA’ cluster, *P* = 0.017) (Supplementary Table 2, Additional File 1).

### Associations between lifestyle clusters and allergic outcomes

Children in the ‘unhealthier lifestyle & higher MVPA’ cluster did not show significantly different odds for allergic disease symptoms or sensitization compared to those in the ‘healthier lifestyle & lower MVPA’ cluster (Table 4). Similar non-significant associations were observed in explorative analyses between lifestyle clusters and symptomatic sensitization, higher-threshold sensitization (IgE ≥ 0.70 kU/L), polysensitization, and manifestation combinations of allergic disease symptoms (≥ 2 symptoms, all three symptoms) (Supplementary Table 4, Additional File 1).

**Table 4:** Cross-sectional associations between lifestyle clusters and prevalence of allergic conditions in the participating children

	Lifestyle cluster		*n*_model_
	Healthier lifestyle & lower MVPA	Unhealthier lifestyle & higher MVPA	
**Asthma symptoms**			
Cases, *n*/*N* (%)	56/276 (20)	32/155 (21)	
Crude model, OR (95% CI)	ref.	1.02 (0.62, 1.66)	431
Adjusted model, OR (95% CI) ^a^	ref.	0.80 (0.46, 1.37)	408
**Allergic rhinitis symptoms**			
Cases, *n*/*N* (%)	69/275 (25)	42/155 (27)	
Crude model, OR (95% CI)	ref.	1.11 (0.71, 1.73)	430
Adjusted model, OR (95% CI) ^a^	ref.	1.32 (0.77, 2.24)	408
**Eczema symptoms**			
Cases, *n*/*N* (%)	38/276 (14)	16/155 (10)	
Crude model, OR (95% CI)	ref.	0.72 (0.38, 1.32)	431
Adjusted model, OR (95% CI) ^a^	ref.	0.89 (0.43, 1.77)	408
**Sensitized to ≥ 1 inhaled allergen** ^**b**^			
Cases, *n*/*N* (%)	39/99 (39)	19/49 (39)	
Crude model, OR (95% CI)	ref.	0.97 (0.48, 1.96)	148
Adjusted model, OR (95% CI) ^a^	ref.	1.27 (0.53, 3.10)	132
**Sensitized to ≥ 1 food allergen** ^**c**^			
Cases, *n*/*N* (%)	20/99 (20)	8/49 (16)	
Crude model, OR (95% CI)	ref.	0.77 (0.30, 1.80)	148
Adjusted model, OR (95% CI) ^a^	ref.	0.91 (0.30, 2.60)	132

ORs with their 95% CIs were obtained from logistic regression models. The cluster ‘healthier lifestyle & lower MVPA’ was set as the reference

^a^ Adjusted for sex, parental allergy history (both parents, mother alone, father alone, or neither parent had a history of at least one of the following: asthma, pollen or animal allergy, food allergy, or atopic eczema), number of older siblings, age when solids were introduced (in months), age when completely stopped being breastfed (in months), maternal smoking during pregnancy (yes or no), current parental smoking (yes or no), furry pets at home or day care facility (yes or no), BMI Z-scores, and highest educational level in the family (high school or less, college, or bachelor’s degree or postgraduate degree)

^b^ IgE response ≥ 0.35 kU/L to at least one of the following: birch, timothy grass, mugwort, cat, dog, horse, house dust mite (*Dermatophagoides Pteronyssinus*), mold (*Cladosporium Herbarum*)

^c^ IgE response ≥ 0.35 kU/L to at least one of the following: cow’s milk, egg, codfish, wheat, soy, peanut

Abbreviations: CI, confidence interval; ISCOLE, the International Study of Childhood Obesity, Lifestyle and the Environment; MVPA, moderate-to-vigorous physical activity; OR, odds ratio; ref., reference group

## Discussion

In the current cross-sectional study among 9–11-year-old Finnish children, we identified two distinct and stable lifestyle clusters characterized by mixed behaviors, named ‘healthier lifestyle & lower MVPA’ and ‘unhealthier lifestyle & higher MVPA’. These lifestyle clusters did not show significant associations with the prevalence of allergic disease symptoms or sensitization. It is noteworthy that our study introduced a novel approach by using data-driven lifestyle clusters as predictive measures for allergic outcomes.

Previous literature suggests that mixed lifestyle clusters comprising both healthy and unhealthy behaviors are most frequently identified across 5–12-year-old child populations [12]. In our study, the observed mixed cluster solutions resulted from time engaged in MVPA. In general, boys have been found to spend more time in MVPA than girls across European school-aged child populations [29]. Likewise, we found that boys dominated the cluster defined by higher MVPA but otherwise unhealthier behavior (higher screen time, unhealthier diet, shorter sleep), whereas the opposite cluster characteristics were more typical for girls. Similar sex differences, wherein physical activity discriminates in a different direction from other lifestyles, have also been observed in the lifestyle clusters of Flemish 10- to 12-year-old children [30, 31]. As findings across European child populations suggest, our mixed cluster solutions may arise from behavior linked to the children’s sex.

Despite our mixed cluster solutions, screen time and adherence to an unhealthy dietary pattern seemed to be the most prominent cluster features, followed by nightly sleep duration. This finding may suggest that part of our children, predominantly boys, had unfavorable dietary habits in front of screens; TV viewing in particular has been linked to a higher consumption of energy-dense snacks and sugar-sweetened beverages [32, 33]. Sleep, on the other hand, has not often been considered in earlier assessments of 5–12-year-old children’s lifestyle clusters [12]. However, our results add to the evidence that nightly sleep duration may be an integral part of children’s health behavior. We found that shorter sleep co-occurred with unhealthier habits (higher screen time, unhealthier diet), whereas longer sleep duration clustered with healthier behavior (less screen time, healthier diet). Similar interplays between sleep, screen time, and dietary habits have been recognized in earlier studies across preschool-aged to adolescent study populations [34–39]; however, in Portuguese ISCOLE participants, longer sleep duration tended to coexist with unhealthier diet and higher screen time [40]. In our sample, time spent with screen devices might occur at the expense of sleep on average.

We did not observe significant associations between the identified lifestyle clusters and allergic symptoms or sensitization in our sample of 9–11-year-old children. To the best of our knowledge, the current study was the first to examine the associations of data-derived lifestyle clusters with allergic outcomes in children. Previous studies assessing joint associations of lifestyle behaviors applied theory-based indices of adopting healthy lifestyle behavior and showed mixed results [13, 14]. Specifically, a worldwide multicenter cross-sectional study among 6–7-year-old children reported a significant trend toward lower prevalence of allergic diseases with increasing adherence to a Healthy Lifestyle Index, which was based on no parental smoking, child’s healthy BMI, adherence to a Mediterranean diet, high physical activity, and non-sedentary behavior [13]. Another cross-sectional study among 5–11-year-old Greek children showed that adherence to an Obesity-preventive lifestyle score, which was based on nine variables related to dietary habits, physical activity, and sedentary behavior, tended to be inversely associated with asthma prevalence, but the association did not remain significant after adjusting for BMI [14]. The specific lifestyle variables considered between our study and earlier indices varied to some extent, which may at least partially explain the differences in results. Importantly, the earlier indices assessed lifestyle behaviors one-dimensionally, i.e., only from the viewpoint of adopting a healthy lifestyle. In practice, lifestyle behaviors can interact in various ways in children, as demonstrated by our data.

In our sample of school-aged children, the lack of significant associations between lifestyle clusters and allergic symptoms and sensitization was consistent across several allergic outcomes, including the co-occurrence of allergic symptoms and symptomatic sensitization. This was despite the finding that the most prominent lifestyle behaviors distinguishing between children appeared to be screen time and adherence to unhealthy dietary pattern, which was characterized by the consumption of discretionary foods high in salt and fat (e.g., French fries, potato chips, fast foods). High intakes of salt and fat, particularly saturated fat, may promote T-helper 2 cell responses [41, 42] and thus predispose to allergic sensitization. A cross-sectional study among 10–12-year-old Greek children from various regions of Athens found a significant association between frequent consumption of salty snacks and fast foods (e.g., hamburger, pizza, popcorn) and asthma symptoms among those who spent > 2 h/day watching TV or playing video games [43]. A possible explanation of no association in our study might derive from the relatively homogeneous study sample. Compared to ISCOLE children of other 11 countries, Finnish ISCOLE participants scored lowest on unhealthy dietary pattern [9] but had high levels of MVPA on average [11]. Our study sample might hence have adopted healthier lifestyle behaviors in general, which may also explain the mixed cluster features. In addition, the sampling frame of the ISCOLE survey intentionally excluded rural areas [20]. However, in Finnish children, living in an urban area has been associated with a healthier dietary pattern [44] and higher levels of moderate MVPA [45] as compared to living in a rural area, and the prevalence of common risk factors for allergic diseases has also differed between urban and rural children [46]. The previous study finding protective associations between the Healthy Lifestyle Index and allergic disease prevalence comprised children from various geographical areas of the world, which probably ensured sufficient variation in lifestyles and other risk factors [13]. In our study setting, children’s lifestyle behaviors, along with other risk factors, may have exhibited limited variation. This could have resulted in an insufficient gradient to detect meaningful associations with allergic outcomes.

Our findings should be viewed in the light of some limitations. The results should be generalized cautiously due to a relatively homogenous study sample, as discussed above. Although we used a stratified sampling strategy based on indicators of socio-economic status to improve variability, we acknowledge that the Finnish ISCOLE survey sample may not be fully representative, given that 36% of the invited schools declined participation. Our cluster analysis also led to a higher exclusion of boys due to incomplete data, yet we were unable to ascertain whether the lack of data was related to healthiness of lifestyles. It remains hence possible that a sex-related non-respondent bias occurred and reduced lifestyle variation to some extent, given that fewer children, but mainly boys, belonged to the ‘unhealthier lifestyle & higher MVPA’ cluster. In addition, our study comprised relatively modest sample sizes, which might reduce the statistical power of our analyses. However, the current study was hypothesis-generating per se. The ISCOLE survey was not initially designed for measuring allergic outcomes, but our secondary analyses suggest that larger and more representative study samples are needed. Importantly, our cross-sectional design prevents from drawing any conclusions on the temporal relationship between children’s lifestyles and allergic outcomes. Although sensitization rates tend to increase with age in childhood [47, 48], allergic diseases often originate in earlier life [19]. In this regard, we acknowledge that the age of our 9- to 11-year-old study population might not be optimal for evaluating the current cross-sectional associations. Longitudinal designs focusing on lifestyle exposures earlier in childhood may demonstrate a more predictive time window.

Our study also had several strengths. The data on allergic disease symptoms were based on a standardized ISAAC questionnaire [25], while allergic sensitization was objectively determined by specific IgE responses to several common allergens. Although data-driven cluster analysis involved some subjective decision-making, it yielded more pragmatic results reflecting real-world interplay between lifestyle behaviors when compared to theory-based indices. By allowing lifestyle patterns to emerge from the data, this approach avoided prior assumptions that may oversimplify the complexity of these interactions. Moreover, objective data were obtained for accelerometer-measured sleep and MVPA, but children’s self-reports on screen time and food consumption might be affected by recall bias. However, regarding screen time, we would not have been able to objectively record this specific type of sedentary behavior. Also, the FFQ showed acceptable validity against food records [21] and was reliable in identifying dietary patterns [9], and we additionally found that the children showed no difficulties in interpreting what most food groups meant despite their young age [21].

## Conclusions

In the present cross-sectional study, two distinct and stable lifestyle clusters were identified among school-aged Finnish children. However, the lifestyle clusters did not demonstrate significant associations with the prevalence of allergic disease symptoms or sensitization. Our results suggest that modifiable lifestyle factors may not play a significant role in allergic conditions within the examined age group. However, future research should ensure larger and more representative study samples. Also, lifestyle behaviors established in earlier childhood may serve as more credible predictors of allergic outcomes.

## Electronic supplementary material

Below is the link to the electronic supplementary material.

Additional File 1: Additional File 1 includes four supplementary tables

## Data Availability

The datasets generated and/or analysed during the current study are not publicly available due to the privacy of individuals who participated in the study but are available from the corresponding author on reasonable request.

## Abbreviations

BMI: Body mass index
CI: Confidence interval
FFQ: Food frequency questionnaire
IgE: Immunoglobulin E
IQR: Interquartile range
ISAAC: The International Study of Asthma and Allergies in Childhood
ISCOLE: The International Study of Childhood Obesity, Lifestyle and the Environment
MVPA: Moderate-to-vigorous physical activity
OR: Odds ratio
ref.: Reference group
SD: Standard deviation
