# Supplementary material for: Associations of multiple lifestyle behaviors with allergic disease symptoms and sensitization in 9–11-year-old Finnish children
Source: BMC Pediatr. 2024 Nov 19;24:749. doi: 10.1186/s12887-024-05218-8 (PMC11575105; doi:10.1186/s12887-024-05218-8)
Supplement: Supplementary file 1 — Additional File 1: Additional File 1 includes four supplementary tables [file 12887_2024_5218_MOESM1_ESM.pdf]

Additional File 1

Article title: Associations of multiple lifestyle behaviors with allergic disease symptoms and sensitization in 9–11-year-old Finnish children

Authors: Henna Peltonen, Anna Kaarina Kukkonen, Liisa Korkalo, Mikael Fogelholm, Mika J. Mäkelä, Maijaliisa Erkkola, Henna Vepsäläinen

Corresponding author: Henna Peltonen; email: henna.peltonen@helsinki.fi

Content

Supplementary Table 1. Description of the two dietary patterns identified among 9–11-year-old Finnish children participating in the ISCOLE survey (*n*=535).

Supplementary Table 2. Distribution of background characteristics according to the lifestyle cluster memberships among 9–11-year-old Finnish children participating in the cross-sectional ISCOLE sub-study on allergic diseases (2012–2013).

Supplementary Table 3. Prevalence of allergen-specific sensitization (specific IgE ≥0.35 kU/L) in 9–11-year-old Finnish children participating in the ISCOLE sub-study on allergic diseases (*n*=148).

Supplementary Table 4. Cross-sectional associations between lifestyle clusters and prevalence of allergic conditions in 9–11-year-old Finnish children participating in the ISCOLE sub-study on allergic diseases (2012–2013). Explorative analyses.

References.

Supplementary Table 1. Description of the two dietary patterns identified among 9–11-year-old Finnish children participating in the ISCOLE survey (*n*=535). Only food items with absolute loadings of >0.30 are shown. The loadings are indicated in parentheses. Please see Mikkilä et al. (1) for more detailed description.

Unhealthy dietary pattern	Healthy dietary pattern
French fries (0.72)	Dark green vegetables (0.71)
Potato chips (0.71)	Orange vegetables (0.69)
Fast foods (0.69)	Vegetables (0.66)
Fried food (nuggets, fish sticks) (0.61)	Beans, lentils, bean curd, eggs (0.64)
Sugar-sweetened sodas (0.60)	Fruits and berries (0.59)
Diet sodas (0.58)	Wholegrains (0.57)
Ice cream (0.55)	Fish (0.56)
Cakes, pastries, doughnuts (0.55)	Other milk products (0.55)
Sweets (candy/chocolate) (0.50)	Cheese (0.49)
Sports drinks (0.43)	
Eigenvalue: 3.8	Eigenvalue: 3.6
Variance explained: 17%	Variance explained: 16%

Supplementary Table 2. Distribution of background characteristics according to the lifestyle cluster membership among 9–11-year-old Finnish children participating in the cross-sectional ISCOLE sub-study on allergic diseases (2012–2013).

	Primary analytic sample, *n*=431			Secondary analytic sample, *n*=148		
**Characteristics**	**Unhealthier lifestyle & higher MVPA**	**Healthier lifestyle & lower MVPA**	***P***	**Unhealthier lifestyle & higher MVPA**	**Healthier lifestyle & lower MVPA**	***P***
n	155	276		49	99	
**Sex, *n* (%)**						
Boys	**99 (64)**	**97 (35)**	**<0.001**	**30 (61)**	**40 (40)**	**0.017**
Girls	**56 (36)**	**179 (65)**		**19 (39)**	**59 (60)**	
**Number of older biological siblings, *n* (%)**						
0	69 (45)	144 (52)	0.134	16 (33)	49 (49)	0.224
1	48 (31)	84 (30)		18 (37)	29 (29)	
≥2	36 (23)	44 (16)		10 (20)	16 (16)	
Missing	2 (1.3)	4 (1.4)		5 (10)	5 (5.1)	
**BMI Z-score**						
Mean ± SD	0.38 ± 1.1	0.19 ± 1.0	0.068	0.49 ± 0.95	0.25 ± 1.1	0.192
Missing, *n* (%)	0 (0.0)	1 (0.36)		0 (0.0)	0 (0.0)	
**Highest educational level in the family, *n* (%)**						
High school or less	**54 (35)**	**63 (23)**	**0.022**	13 (27)	22 (22)	0.597
College degree	**42 (27)**	**84 (30)**		11 (22)	22 (22)	
Bachelor's or postgraduate degree	**58 (37)**	**129 (47)**		20 (41)	52 (53)	
Missing	1 (0.65)	0 (0.0)		5 (10)	3 (3.0)	
**Furry pets at home or daycare facility, *n* (%)**						
Yes	72 (46)	120 (43)	0.577	20 (41)	33 (33)	0.226
Missing	1 (0.65)	3 (1.1)		5 (10)	4 (4.0)	
**Age at introducing solids, months**						
Median (IQR)	4 (3–4.5)	4 (3–5)	0.961[a]	4 (3–4)	4 (3–5)	0.339[a]
Missing, *n* (%)	6 (3.9)	11 (4.0)		5 (10)	11 (11)	
**Age completely stopped being breastfed, months**						
Median (IQR)	6 (3–11)	7 (4–11)	0.247[a]	10 (6–12)	8 (4–12)	0.335[a]
Missing, *n* (%)	3 (1.9)	6 (2.2)		5 (10)	7 (7.1)	

Supplementary Table 2. (Continued)

**Parental history of allergic disease [b], *n* (%)**						
Neither parent	61 (39)	119 (43)	0.234	19 (39)	34 (34)	0.667
Father alone	33 (21)	44 (16)		10 (20)	18 (18)	
Mother alone	41 (26)	64 (23)		8 (16)	24 (24)	
Both parents	19 (12)	49 (18)		7 (14)	19 (19)	
Missing	1 (0.65)	0 (0.0)		5 (10)	4 (4.0)	
**Current parental smoking [c], *n* (%)**						
Yes	38 (25)	48 (17)	0.070	10 (20)	16 (16)	0.408
Missing	1 (0.65)	0 (0.0)		5 (10)	4 (4.0)	
**Maternal smoking during pregnancy, *n* (%)**						
Yes	11 (7.1)	11 (4.0)	0.160	2 (4.1)	3 (3.0)	0.658[d]
Missing	2 (1.3)	4 (1.4)		5 (10)	7 (7.1)	

P-values are based on independent samples t-tests for continuous variables and chi-squared independence tests for categorical variables unless otherwise indicated. Significant differences between clusters (P<0.05) are indicated in boldface. Missing observations are shown if they were present.

[a] based on Mann-Whitney U-test

[b] at least one of the following: asthma, pollen or animal allergy, food allergy, or atopic eczema

[c] one or both of the parents

[d] based on Fisher's exact test

Abbreviations: IQR, inter-quartile range (25th to 75th percentile); ISCOLE, the International Study of Childhood Obesity, Lifestyle and the Environment; SD, standard deviation.

Supplementary Table 3. Prevalence of allergen-specific sensitization (specific IgE ≥0.35 kU/L) in 9–11-year-old Finnish children participating in the ISCOLE sub-study on allergic diseases (*n*=148). The table presents sensitization rates to common inhaled and food allergens, including their components.

	**Specific IgE ≥0.35 kU/L, *n* (%)**
**Inhaled allergen**	
Birch pollen	41 (28)
Bet v 1	36 (24)
Timothy grass	33 (22)
Mugwort	17 (11)
Cat dander	36 (24)
Dog dander	30 (20)
Horse dander	18 (12)
House dust mite [a]	11 (7.4)
Mold [b]	5 (3.4)
**Food allergen**	
Cow's milk	15 (10)
Egg white	15 (10)
Ovomucoid	3 (2.0)
Ovalbumin	10 (6.8)
Peanut	13 (8.8)
Ara h 2	2 (1.4)
Ara h 8	32 (22)
Codfish	0 (0.0)
Soy	8 (5.4)
Gly m 4	31 (21)
Gly m 5	0 (0.0)
Gly m 6	1 (0.68)
Wheat	8 (5.4)
Tri a 14	0 (0.0)
Omega-5 gliadin	0 (0.0)

[a] *Dermatophagoides Pteronyssinus*

[b] *Cladosporium Herbarum*

Abbreviations: IgE, immunoglobulin E; ISCOLE, the International Study of Childhood Obesity, Lifestyle and the Environment.

Supplementary Table 4. Cross-sectional associations between lifestyle clusters and prevalence of allergic conditions in 9–11-year-old Finnish children participating in the ISCOLE sub-study on allergic diseases (2012–2013). Explorative analyses.

	Lifestyle cluster		
**Explorative outcomes**	*Healthier lifestyle & lower MVPA*	*Unhealthier lifestyle & higher MVPA*	n_{model}
**≥2 allergic disease symptoms** [a]			
Cases, *n*/*N* (%)	40/275 (15)	20/155 (13)	
Crude model, OR (95% CI)	ref.	0.87 (0.48, 1.54)	430
Adjusted model, OR (95% CI) [b]	ref.	0.84 (0.43, 1.61)	408
**Three allergic disease symptoms** [a]			
Cases, *n*/*N* (%)	11/275 (4.0)	7/155 (4.5)	
Crude model, OR (95% CI)	ref.	1.14 (0.41, 2.95)	430
Adjusted model, OR (95% CI) [b]	ref.	1.27 (0.41, 3.72)	408
**IgE ≥0.70 kU/L to any inhaled allergen** [c]			
Cases, *n*/*N* (%)	36/99 (36)	19/49 (39)	
Crude model, OR (95% CI)	ref.	1.11 (0.54, 2.24)	148
Adjusted model, OR (95% CI) [b]	ref.	1.42 (0.60, 3.41)	132
**IgE ≥0.70 kU/L to any food allergen** [d]			
Cases, *n*/*N* (%)	16/99 (16)	5/49 (10)	
Crude model, OR (95% CI)	ref.	0.59 (0.18, 1.62)	148
Adjusted model, OR (95% CI) [b]	ref.	0.73 (0.20, 2.39)	132
**Symptomatic sensitization** [e]			
Cases, *n*/*N* (%)	32/95 (34)	14/44 (32)	
Crude model, OR (95% CI)	ref.	0.92 (0.42, 1.95)	139
Adjusted model, OR (95% CI) [b]	ref.	1.22 (0.48, 3.13)	132
**Polysensitization** [f]			
Cases, *n*/*N* (%)	34/99 (34)	17/49 (35)	
Crude model, OR (95% CI)	ref.	1.02 (0.49, 2.07)	148
Adjusted model, OR (95% CI) [b]	ref.	1.17 (0.46, 2.98)	132

ORs with their 95% CIs were obtained from logistic regression models. The cluster ‘healthier lifestyle & lower MVPA’ was set as the reference.

[a] asthma, allergic rhinitis, eczema.

[b] adjusted for sex, parental allergy history (both parents, mother alone, father alone, or neither parent had a history of at least one of the following: asthma, pollen or animal allergy, food allergy, or atopic eczema), number of older siblings, age when solids were introduced (in months), age when completely stopped being breastfed (in months), maternal smoking during pregnancy (yes or no), current parental smoking (yes or no), furry pets at home or day care facility (yes or no), BMI Z-scores, and highest educational level in the family (high school or less, college, or bachelor’s degree or postgraduate degree).

[c] birch, timothy grass, mugwort, cat, dog, horse, house dust mite (*Dermatophagoides Pteronyssinus*), mold (*Cladosporium Herbarum*).

[d] cow’s milk, egg, codfish, wheat, soy, peanut.

[e] sensitization to any allergen (specific IgE ≥0.35 kU/L) accompanied by symptoms of any allergic disease (asthma, allergic rhinitis, eczema).

[f] sensitization to at least two allergens (specific IgE ≥0.35 kU/L to any two or more inhaled or food allergens).

Abbreviations: CI, confidence interval; IgE, immunoglobulin E; ISCOLE, the International Study of Childhood Obesity, Lifestyle and the Environment; MVPA, moderate-to-vigorous physical activity; OR, odds ratio; ref., reference group.

Reference

1. Mikkilä V, Vepsäläinen H, Saloheimo T, Gonzalez SA, Meisel JD, Hu G, et al. An international comparison of dietary patterns in 9–11-year-old children. Int J Obes Suppl 2015;5(Suppl 2):S17–21. doi:10.1038/ijosup.2015.14
